# ArraySolver: An Algorithm for Colour-Coded Graphical Display and Wilcoxon Signed-Rank Statistics for Comparing Microarray Gene Expression Data

**DOI:** 10.1002/cfg.369

**Published:** 2004-02

**Authors:** Haseeb Ahmad Khan

**Affiliations:** Research Centre Armed Forces Hospital T-835 PO Box 7897 Riyadh 11159 Saudi Arabia

## Abstract

The massive surge in the production of microarray data poses a great challenge for proper analysis and interpretation. In recent years numerous computational tools have been developed to extract meaningful interpretation of microarray gene expression data. However, a convenient tool for two-groups comparison of microarray data is still lacking and users have to rely on commercial statistical packages that might be costly and require special skills, in addition to extra time and effort for transferring data from one platform to other. Various statistical methods, including the *t*-test, analysis of variance, Pearson test and Mann–Whitney U test, have been reported for comparing microarray data, whereas the utilization of the Wilcoxon signed-rank test, which is an appropriate test for two-groups comparison of gene expression data, has largely been neglected in microarray studies. The aim of this investigation was to build an integrated tool, ArraySolver, for colour-coded graphical display and comparison of gene expression data using the Wilcoxon signed-rank test. The results of software validation showed similar outputs with ArraySolver and SPSS for large datasets. Whereas the former program appeared to be more accurate for 25 or fewer pairs (*n* ≤ 25), suggesting its potential application in analysing molecular signatures that usually contain small numbers of genes. The main advantages of ArraySolver are easy data selection, convenient report format, accurate statistics and the familiar Excel platform.


**Comparative and Functional Genomics**
*Comp Funct Genom* 2004; **5**: 39–47.
Published online in Wiley InterScience (www.interscience.wiley.com). **DOI:** 10.1002/cfg.369

**Short Communication**

### ArraySolver: an algorithm for colour-coded graphical display and Wilcoxon signed-rank statistics for comparing microarray gene expression data

Haseeb Ahmad Khan*
*Research Centre, Armed Forces Hospital, Riyadh, Saudi Arabia*

**Correspondence to:*
*Haseeb Ahmad Khan, Research Centre, Armed Forces Hospital, T-835, PO Box 7897, Riyadh 11159, Saudi Arabia.*
*E-mail: khan_haseeb@yahoo.com*

Received: 7 April 2003
Revised: 22 November 2003
Accepted: 27 November 2003

### Abstract

**The massive surge in the production of microarray data poses a great challenge for proper analysis and interpretation. In recent years numerous computational tools have been developed to extract meaningful interpretation of microarray gene expression data. However, a convenient tool for two-groups comparison of microarray data is still lacking and users have to rely on commercial statistical packages that might be costly and require special skills, in addition to extra time and effort for transferring data from one platform to other. Various statistical methods, including the *t*-test, analysis of variance, Pearson test and Mann–Whitney U test, have been reported for comparing microarray data, whereas the utilization of the Wilcoxon signed-rank test, which is an appropriate test for two-groups comparison of gene expression data, has largely been neglected in microarray studies. The aim of this investigation was to build an integrated tool, ArraySolver, for colour-coded graphical display and comparison of gene expression data using the Wilcoxon signed-rank test. The results of software validation showed similar outputs with ArraySolver and SPSS for large datasets. Whereas the former program appeared to be more accurate for 25 or fewer pairs ($n \leq 25$), suggesting its potential application in analysing molecular signatures that usually contain small numbers of genes. The main advantages of ArraySolver are easy data selection, convenient report format, accurate statistics and the familiar Excel platform. Copyright © 2004 John Wiley & Sons, Ltd.**

**Keywords:** gene expression; microarray; colour-coded display; statistical comparison; Wilcoxon signed-rank test; software

### Introduction

Microarray is a versatile technique for measuring the expression of thousands of genes simultaneously in a single experiment. However, capturing the hidden treasure from huge microarray datasets is a great challenge for scientists. The primary microarray data need to be normalized to correct for slide-to-slide experimental variation before any statistical interpretation can be meaningfully carried out (Hoffmann *et al.*, 2002; Smid-Koopman *et al.*, 2000). One of the major goals of microarray data analysis is the identification of genes that are differentially expressed within two or more kinds of samples or experimental conditions. Both parametric and non-parametric approaches have been applied for this purpose (Thomas *et al.*, 2001; Zhao and Pan 2003; Troyanskaya *et al.*, 2002). Tusher *et al.* (2001) have developed an Excel-based algorithm known as SAM (significance analysis of microarrays) for detection of differentially expressed genes between groups of samples. On the other hand, gene clustering (hierarchical grouping) is a commonly used computational tool for molecular classification of disease states, functional grouping of genes and biological

Copyright © 2004 John Wiley & Sons, Ltd.

description of gene regulation (Wang *et al.*, 1999; Golub *et al.*, 1999; Gaasterland and Bekiranov 2000; Tamayo *et al.*, 1999). Usually the strategies of filtering differentially expressed genes and functional clustering are applied in tandem for molecular classification of gene signatures or fingerprints with embedded diagnostic and prognostic features (Alizadeh *et al.*, 2000; Ladanyi *et al.*, 2001; Ahr *et al.*, 2002; Mycko *et al.*, 2003; Xu *et al.*, 2002).

The usage of an appropriate statistical method for two-group comparisons (e.g. normal vs. diseased) is an important criterion for effective application of gene signatures. The cluster analysis cannot be considered a valid method for comparing gene expression between the two samples or groups (Thomas *et al.*, 2001). Similarly, the tools for determining differentially expressed genes tend to apply filters that would disturb the basic configuration of gene signatures and would not be suitable for an integrated two-group comparison. Recently, a wide range of statistical procedures including the t-test (Notterman *et al.*, 2001; Tanaka *et al.*, 2000; Zhou *et al.*, 2001), analysis of variance (Maxwell *et al.*, 2002; Bushel *et al.*, 2002), Pearson correlation (Bouras *et al.*, 2002), Welch test (Han *et al.*, 2003) and Mann–Whitney U test (Kihara *et al.*, 2001; Rus *et al.*, 2002) have been used for comparison of microarray data. Some investigators have also chosen *xy*-scatter plots for pairwise visual comparison of microarray expression data (Wang *et al.*, 1999; Smid-Koopman *et al.*, 2000).

Although normalization of microarray data might validate parametric statistics for detecting differences between the two groups, a non-parametric (distribution-free) approach seems to be more reliable and appropriate statistics for such a data structure. The Mann–Whitney U test (Wilcoxon rank sum test) is an important non-parametric test and has been used for testing significance between two groups in microarray studies (Kihara *et al.*, 2001; Rus *et al.*, 2002). This test is identical to independent sample $t$-test in a parametric setting and is valid for testing differences between independent groups. The Wilcoxon matched-pairs signed-rank test (the counterpart of the parametric paired $t$-test) examines the differences between dependent groups (Wilcoxon 1945, 1947; Siegel 1956), and could be more useful for analysing microarray expression data. The Wilcoxon signed-rank test has been applied to pairwise comparison of gene expression data obtained from reverse-transcription PCR (Beenken *et al.*, 2002; Yu *et al.*, 2001; Leygue *et al.*, 1999), real-time PCR (Pfaffl *et al.*, 2003), *in situ* hybridization (Robinson *et al.*, 2002), immunohistochemistry (Johnston *et al.*, 1995) and laser dosimetry (Bradbury *et al.*, 1994), whereas the potential application of the Wilcoxon signed-rank test has largely been neglected for microarray data analysis, possibly due to the computational complexities, especially when the number of pairs is large (Campbell and Machin, 1996; Efron and Tibshirani, 2002).

The objective of this study was to develop a Microsoft Excel-based tool for minimizing the complexities of gene expression data by using colour-coded graphics and to perform the Wilcoxon signed-rank test within the same framework.

### Methods

### Software design

The ArraySolver program has been developed in Microsoft Excel (Version 2000) on a Pentium III computer. The program is mainly composed of two worksheets, one for data entry and the other for report display. Two additional worksheets are also used for statistical computations but the user has no interaction with them.

### Data entry window

This is essentially an Excel worksheet, with the availability of four controls, including two option buttons and two command buttons. The user has to select one of the option buttons to specify whether all the selected genes or only differentially expressed genes (ratio $\geq 1.5$ or $\leq 0.5$) will be used for visual display and analysis. The two command buttons, 'Display multi-columns' and 'Compare 2-columns' are used to execute the program; the former is meant for visual display of multiple groups together and the latter for display of two groups and their statistical comparison. The columns in the worksheet specify different groups and the rows indicate individual genes in the microarray. The top row is considered as a header row (column titles); if the title row is absent, a blank row should be inserted at the top of the worksheet for accurate results. The data can be

Copyright © 2004 John Wiley & Sons, Ltd. 

directly keyed-in or alternatively imported from an external source.

### Report window

The colour-coded expression profiles are displayed on a new Excel worksheet (Figures 1 and 2). There are two buttons, 'Next' and 'Wilcoxon test', on the report window; the former is used to display the data-entry window and the latter to perform a Wilcoxon signed-rank test. The graphic output of gene expression data is a collection of colour-coded squares, either spanning horizontally left to right (10 squares in each row) and expanding vertically downwards (2-groups mode), or arranged in vertical columns (multi-groups mode). The gene expression values have been classified into seven categories (different colour-codes), three for down-regulation (light to dark blue), three for upregulation (light to dark red) and one for norm-regulation (grey).

### Procedure for creating visual arrays

Following the data entry in the worksheet, the program is executed by clicking the appropriate command button. The selection of data is interactive and controlled by input boxes. The colour-coded graphical output is displayed on the report window.

### Differentially expressed genes

It is anticipated that comparing whole arrays using ArraySolver may not be very sensible in many cases for two reasons. First, normalization can lead to microarrays with the same or very similar overall average expression. Second, very large sample sizes give the tests enormous power to detect changes; this makes findings statistically significant yet scientifically uninteresting. Thus, the gene expression ratios $\geq 1.5$ and $\leq 0.5$ were set to extract up- and downregulated genes, respectively. This simple fold-change procedure was primarily aimed for screening useful information from large datasets. Similar cut-off ratios have been reported in various microarray studies (Okabe *et al.*, 2001; Gutgemann *et al.*, 2001; Wang *et al.*, 2001; Bull *et al.*, 2001). However, if a different cut-off scale is intended or any other computing procedure is desired, it should be performed before transferring the data to ArraySolver. SAM is one of the useful software packages that is publicly available as an Excel 'add-in' for the identification of differentially expressed genes.

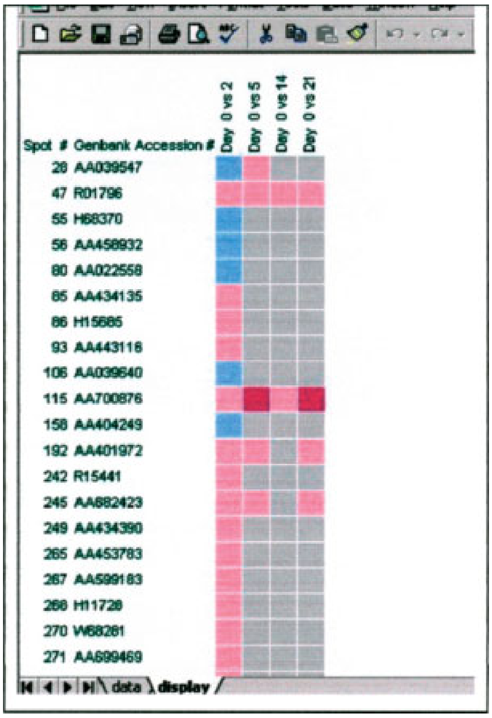

**Figure 1.** Display window showing colour-coded gene expression of multiple groups. The data in columns 1, 2, 4, 5, 6 and 7 of the original data file (Mariadason *et al.*, 2002) were transferred to ArraySolver. The program was executed by clicking 'Display multi-columns' after choosing the option of differentially expressed genes (the extreme left column with expression data is set as base) and selecting the entire data (13 638 genes). The group 'Day 0 vs. 2' is showing differentially expressed genes (partial view of 1451 genes), whereas the remaining three groups show their expression colour-codes for the base genes

### Wilcoxon signed-rank test

ArraySolver has an in-built capability for the Wilcoxon signed-rank test without any link to an external statistical package. The algorithm for statistical comparison has been developed according to the standard methodology (Wilcoxon 1945, 1947; Siegel 1956). Although the computations are straightforward, the procedure tends to be complex due to the involvement of two types of ties. In the first tie, the two scores of any pair are equal [difference $(d) = 0$] and such pairs have to be dropped from the analysis. The second type of tie

Copyright © 2004 John Wiley & Sons, Ltd. 

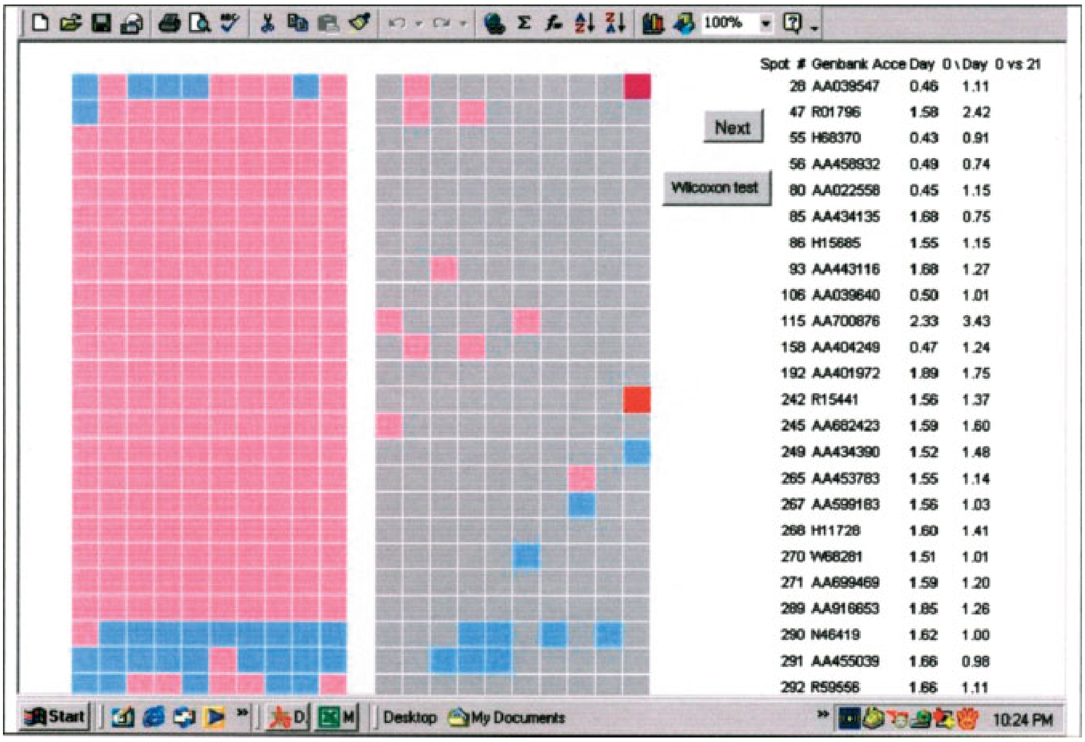

**Figure 2.** Display window showing colour-coded gene expression of two groups. The data in columns 1, 2, 4 and 7 of the original data file (Mariadason *et al.*, 2002) were transferred to ArraySolver. The program was executed by clicking 'Compare 2-columns' after choosing the option of differentially expressed genes (left graphical panel). Clicking the 'Next' button prompts the user for next selection, whereas clicking 'Wilcoxon test' computes the $p$ value for the data currently displayed occurs when two or more $d$s have identical values, and thus the same ranks (average of the individual ranks) should be assigned to them. The first tie was managed using the Excel feature 'filter', whereas a special 'on-off switch' based on cell formatting (red colour for switch-on and green for switch-off) was developed to detect the sets of identical $d$s within the selected data range and the assignment of average ranks (Figure 3). The computations including pair differences, tie adjustments, rank allocation and sign restoration are performed on a separate sheet and the final results are transferred to the display window, so that the user just has to click a single button to get the results of the statistical comparisons.

### Results and discussion

The data from Mariadason *et al.*'s (2002) study were used to validate the optimal functioning of ArraySolver. The compressed Excel file containing normalized expression data for 13 638 genes was downloaded from the website. The data in column 1 (Spot No.), column 2 (GenBank Accession No.) and columns 4, 5, 6 and 7 (day 0 vs. days 2, 5, 14 and 21, respectively) were copied to the worksheet of ArraySolver. In this study, the groups representing columns 4–7 of the original data file have been renamed as groups 1, 2, 3 and 4, respectively, for the sake of simplicity. The colour-coded display of expression profiles from multiple samples (Figure 1) and 2-samples (Figure 2) clearly indicate that ArraySolver efficiently converts numeric gene expression data into two convenient graphical formats for better visualization. Recently, Schageman *et al.* (2002) have also used Excel to develop a tool for visual interpretation of microarray data. However, the graphical output with the use of ArraySolver is a typical prototype of microarray signals, in contrast to colour-coded scatter plots reported earlier (Schageman *et al.*, 2002).

Copyright © 2004 John Wiley & Sons, Ltd. 

```
Set currentcell = Worksheets("stat1").Range("D1")
Do  Until currentcell. Value = ""
  Set nextcell = currentcell.Offset(1,0)
   If nextcell. Value = currentcell.Value Then
     currentcell.Interior.ColorIndex = 3
     nextcell.Interior.ColorIndex = 4
    End If
  Set currentcell = nextcell
 Loop
-----------------------------------
Function myaverage( )
  s = Application. WorksheetFunction.Count_
          (Sheets("stat1").Range("d:d"))
  With Sheets("stat1")
    For u = 1 To s + 1
      For v = 2 To s + 1
        If .Cells(u, 4).Interior.ColorIndex = 3 Then
          u = u
        If .Cells(v, 4).Interior.ColorIndex = 4 Then
          myrange = .Range(.Cells(u, 5), .Cells(v, 5))
          .Range(.Cells(u, 5), .Cells(v, 5)).Value = _
          Application.WorksheetFunction. _
           Average(myrange)
          .Range(.Cells(u, 4), .Cells(v, 4)).Interior. _
          ColorIndex = 2
        End If
        End If
      Next v
    Next u
  End With
End Function
```

**Figure 3.** The upper panel shows the coding of 'on–off switch' for selective detection of identical cells. The function 'myaverage' (lower panel) works in conjunction with the switch for the assignment of average ranks

To ascertain the accuracy of the Wilcoxon statistics, $z$ and $p$ values for three different pairs and variable numbers of genes (20–13 638) were computed by ArraySolver and SPSS (SPSS for Windows) for a comparative assessment. Both the $z$ scores and $p$ values obtained from ArraySolver and SPSS were identical for all the comparisons except for those with fewer pairs ($n = 20$) (Table 1). This difference is due to the fact that SPSS computes Wilcoxon's $p$ values using $z$ scores irrespective of the number of pairs, whereas ArraySolver strictly follows the standard procedure (Wilcoxon, 1945, 1947; Siegel, 1956) and relies on $T$ scores when the total number of pairs is 25 or less. In fact, the original Wilcoxon table cannot be used if $n > 25$; however, it has been shown that in such cases the sum of the ranks ($T$) is practically normally distributed (Siegel, 1956). Thus, the observed $z$ computed from $T$ scores [$z = (T - \mu_T)/\sigma_T$] is also normally distributed with zero mean and unit variance and provides an excellent large-sample approximation.

In the ArraySolver program, two sets of $p$ values are pre-stored; one set for matching $z$ scores with the corresponding $p$ value (for $n > 25$) and the other for matching $T$ scores with the respective $p$ values for $n \leq 25$. Although ArraySolver and SPSS are equally efficient for $n > 25$, the former program is more reliable for small datasets and therefore would be more useful for comparing data from studies with fewer genes, such as molecular signatures (Su *et al.*, 2001; Ramaswamy *et al.*, 2003).

Another advantage of ArraySolver is the flexible mode of data selection, which is performed by a single mouse click at the top of column (entire column selection) or by mouse dragging (specified range selection). Unfortunately, the range selection feature is not available in SPSS, hence copy–paste of the intended data range to new columns has to be performed prior to data analysis with this package. SPSS is one of the most powerful and versatile statistical packages, developed for a wide

**Table 1.** Wilcoxon signed-rank statistics for comparing two groups using SPSS and ArraySolver

| Number of genes* | Groups | SPSS $z$ | SPSS $p$ (2-tail) | ArraySolver $z$ or ($T$) | ArraySolver $p$ (2-tail) |
|---|---|---|---|---|---|
| 13 638 | 1 vs. 2 | −0.030 | 0.976 | −0.029 | 0.9760 |
| | 1 vs. 3 | −10.178 | 0.000 | −10.177 | 0.0000 |
| | 1 vs. 4 | −9.191 | 0.000 | −9.191 | 0.0000 |
| 1000 | 1 vs. 2 | −11.719 | 0.000 | −11.719 | 0.0000 |
| | 1 vs. 3 | −20.516 | 0.000 | −20.516 | 0.0000 |
| | 1 vs. 4 | −22.418 | 0.000 | −22.418 | 0.0000 |
| 100 | 1 vs. 2 | −3.658 | 0.000 | −3.658 | 0.0003 |
| | 1 vs. 3 | −2.654 | 0.008 | −2.654 | 0.0080 |
| | 1 vs. 4 | −1.564 | 0.118 | −1.564 | 0.1188 |
| 20 | 1 vs. 2 | −2.427 | **0.015** | 40 ($T$) | 0.0200 |
| | 1 vs. 3 | −0.784 | 0.433 | 84 ($T$) | >0.050 |
| | 1 vs. 4 | −2.165 | **0.030** | 47 ($T$) | 0.0500 |

Both SPSS and ArraySolver show similar results for large sample sizes, whereas for $n = 20$ the $p$ values obtained from SPSS are lower (shown in bold) than the $p$ values from ArraySolver. This difference is due to the fact that SPSS computes $p$ values using $z$ scores irrespective of number of pairs, whereas ArraySolver uses $T$ scores instead of $z$ for computing $p$ values when the sample pairs are 25 or less ($n \leq 25$). Groups 1, 2, 3 and 4 in this table represent the groups stored in columns 4, 5, 6 and 7 of the original Excel file of microarray expression data of Mariadason's study (Mariadason *et al.*, 2002); data used with his kind permission.

* Number count starts from the first gene in the column till the specified number. The total number of genes in the microarray data = 13 638.

Copyright © 2004 John Wiley & Sons, Ltd. 

range of applications. ArraySolver, on the other hand, has been specifically designed for display and analysis of microarray gene expression data. The selection of a Microsoft Excel spreadsheet for the development of ArraySolver was based on the fact that Excel provides excellent computational and visualization power for robust analysis of microarray data (Convey *et al.*, 2002; Schageman *et al.*, 2002). The Excel platform of ArraySolver can also be used for data normalization prior to statistical evaluation, and this integrated approach would significantly minimize the time and effort in transferring data from one program to another for specific purposes (Mariadason *et al.*, 2002; Bull *et al.*, 2001).

The appropriateness of various parametric and non-parametric tests, including the Wilcoxon signed-rank test, Mann–Whitney U test, independent sample $t$-test, paired $t$-test and Pearson test for two-sample comparison of microarray expression data was also studied. Two datasets, $n = 20$ (first 20 genes) and $n = 13\,638$ (total genes in the same data file) were subjected to statistical comparisons between three different pairs using SPSS (Table 2). For the large dataset, all the parametric and non-parametric tests appeared to be similar except for comparing group 1 with group 2, where both the Wilcoxon signed-rank test ($p = 0.976$) and the Mann–Whitney U test ($p = 0.912$) showed similar results that were totally different from the other tests ($p = 0.000$). This disparity could, to some extent, be explained with the help of histograms showing the frequency distribution for these two groups (Figure 4). The Wilcoxon statistics between group 1 (day 0 vs. day 2) and group 2 (day 0 vs. day 5) resulted in 7265 negative ranks (mean rank = 6402; sum of ranks = 4.7E07) and 6373 positive ranks (mean = 7294; sum = 4.6E07) with a $z$ score of $-0.03$, whereas the minimum $z$ scores of $\pm 1.65$ (1-tail) and $\pm 1.96$ (2-tail) are required to reject the null hypothesis at $p < 0.05$. On the other hand, a large sample size and normalized data format have seemingly favoured the highly significant output with parametric tests (Table 2). For small datasets, all the statistical methods (except the Pearson test) resulted in a similar pattern, although their respective $p$ values were not identical (Table 2). In fact, the Pearson test is most suitable for correlation studies and may not have such a potential for pairwise comparisons. Although parametric tests can be used for data normalization or identification of differentially expressed genes, the Wilcoxon signed-rank test would be a safer and more robust choice for microarray data analysis (Liu *et al.*, 2002). Notwithstanding their conservativeness, or having a lower statistical power with normalized data (Thomas *et al.*, 2001), non-parametric tests have been suggested to be more advantageous when the computationally identified genes need to be tested biologically (Troyanskaya *et al.*, 2002).

Finally, an attempt was made to describe the real application of ArraySolver (Table 3) using earlier published data (Mariadason *et al.*, 2002). The Excel file of normalized array data of 2286 genes that were differentially expressed during

**Table 2.** Comparative view of 2-tailed significance levels for small and large datasets using various statistical methods*

| Number of genes | Groups | Wilcoxon signed-rank test | Mann–Whitney U test | Independent t-test | Paired t-test | Pearson test |
|---|---|---|---|---|---|---|
| 13 638 | 1 vs. 2 | 0.976 | 0.912 | 0.000 | 0.000 | 0.000 |
| | 1 vs. 3 | 0.000 | 0.000 | 0.000 | 0.000 | 0.000 |
| | 1 vs. 4 | 0.000 | 0.000 | 0.000 | 0.001 | 0.001 |
| 20 | 1 vs. 2 | 0.015 | 0.003 | 0.002 | 0.014 | 0.034 |
| | 1 vs. 3 | 0.433 | 0.358 | 0.406 | 0.334 | 0.195 |
| | 1 vs. 4 | 0.030 | 0.020 | 0.035 | 0.017 | 0.205 |

For large datasets, almost all the statistical methods showed similar output except the 1 vs. 2 groups comparison showed a huge difference between other tests ($p = 0.000$) and Wilcoxon signed-rank (0.976) or Mann–Whitney U test (0.912). For small datasets, the significant/non-significant pattern (not the $p$ values) was similar with all the statistical methods except the Pearson test. Details about groups and number of genes have been given in footnote of Table 1. Refer to Figure 4 for additional information on data structure.

* SPSS was used for all the statistical tests reported in this table.

Copyright © 2004 John Wiley & Sons, Ltd. 

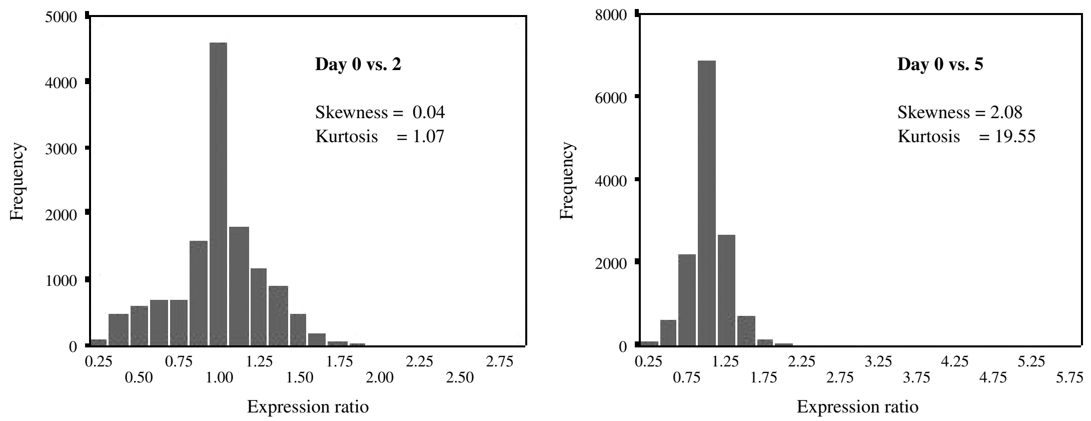

**Figure 4.** Histograms showing frequency distribution of all the 13 638 genes for group 1 (day 0 vs. 2) and group 2 (day 0 vs. 5) towards explaining the statistical variations observed among various parametric and non-parametric tests while comparing these two groups (Table 2). Lower values of skewness and kurtosis for group 1 indicate a symmetrical distribution with a flat top near the mean. In contrast, the frequency distribution for group 2 suggests deviation from normal distribution due to lack of symmetry (high skewness) and heavy tailing (high kurtosis). Note: lower frequencies may not be visible on *x* axis due to large scaling of *y* axis

**Table 3.** Application of ArraySolver in time-course statistical evaluation of functionally characterized gene-subsets on maturation of colon carcinoma cell lines*

| Functional group | Genes (*n*) | *p* values originally reported* | *p* values for each time point using ArraySolver Day 0 vs. following days: Day 2 | Day 5 | Day 14 | Day 21 |
|---|---|---|---|---|---|---|
| Cell cycle | 38 | <0.0001 | 0.1676 | 0.0000 | 0.0000 | 0.0000 |
| DNA synthesis/repair | 59 | <0.0001 | 0.0120 | 0.0000 | 0.0000 | 0.0000 |
| ESTs | 948 | <0.0001 | 0.0000 | 0.0000 | 0.0001 | 0.0000 |
| Kinases/phosphatases | 85 | <0.0001 | 0.0014 | 0.0340 | 0.0198 | 0.0182 |
| Protein processing | 53 | <0.0001 | 0.5686 | 0.1236 | 0.2460 | 0.0040 |
| Drug metabolism | 34 | <0.0001 | 0.9680 | 0.1528 | 0.0024 | 0.0010 |

* Data represented here are part of Table 1 from originally published report by Mariadason *et al.* (2002) and used with his kind permission. Although the overall significance in the original study (column 3, all *p* values < 0.0001) appeared to be same, a typical pattern observed by ArraySolver might help to understand the role of various genes functionally involved in the maturation phase of Caco-2 colon carcinoma cell lines. *n*, number of genes with altered expression; EST, expressed sequence tag.

Caco-2 cell differentiation was kindly provided by Professor J. M. Mariadason. We selected six of the 25 predefined functional categories and the filtered data were transferred to ArraySolver for a time-course assessment of these functional groups on Caco-2 cell maturation and differentiation, with respect to their overall effect reported earlier (Mariadason *et al.*, 2002). It was presumed that a time-course strategy of testing significance levels would be more realistic than the overall significance of a particular gene set defining a functional group. The resulting output, in the form of typical patterns of *p* values, might be helpful in discovering new insights explaining the exact molecular pathways of Caco-2 cell maturation (Table 3).

In conclusion, ArraySolver is a convenient tool for analysis and interpretation of gene expression data. The facility of colour-coded graphical display minimizes the complexity of tabular data, whereas the Wilcoxon signed-rank test provides an appropriate and reliable statistical analysis. Although ArraySolver can handle very large datasets, it is highly desirable to apply this software to pre-filtered data or to gene signatures for meaningful interpretation of the results.

Copyright © 2004 John Wiley & Sons, Ltd. 

### Availability of software

To obtain the software, contact the author by E-mail: **khan_haseeb@yahoo.com**

### Acknowledgements

The author is extremely grateful to Professor John M. Mariadason, Department of Oncology, Albert Einstein College of Medicine, New York, USA, for his kind permission to utilize his microarray data for the validation of the Array-Solver program.

### References

Ahr A, Karn T, Solbach C, *et al.* 2002. Identification of high-risk breast-cancer patients by gene expression profiling. *Lancet* **359**: 131–132.

Alizadeh AA, Eisen MB, Davis RE, *et al.* 2000. Distinct types of diffuse large B-cell lymphoma identified by gene expression profiling. *Nature* **403**: 503–511.

Beenken SW, Hockett R Jr, Grizzle W, *et al.* 2002. Transforming growth factor-$\alpha$: a surrogate endpoint biomarker. *J Am Coll Surg* **195**: 149–158.

Bouras T, Southey MC, Chang AC, *et al.* 2002. Stanniocalcin 2 is an estrogen-responsive gene coexpressed with the estrogen receptor in human breast cancer. *Cancer Res* **62**: 1289–1295.

Bradbury AW, Carter DC, Miller WR, Cho-Chung YS, Clair T. 1994. Protein kinase A (PK-A) regulatory subunit expression in colorectal cancer and related mucosa. *Br J Cancer* **69**: 738–742.

Bull JH, Ellison G, Patel A, *et al.* 2001. Identification of potential diagnostic markers of prostate cancer and prostatic intraepithelial neoplasia using cDNA microarray. *Br J Cancer* **84**: 1512–1519.

Bushel PR, Hamadeh HK, Bennett L, *et al.* 2002. Computational selection of distinct class- and subclass-specific gene expression signatures. *J Biomed Inform* **35**: 160–170.

Campbell MJ, Machin D. 1996. *Medical Statistics.* Wiley: Chichester; 83.

Conway T, Kraus B, Tucker DL, *et al.* 2002. DNA array analysis in a Microsoft windows environment. *Biotechniques* **110**: 112–119.

Efron B, Tibshirani R. 2002. Empirical Bayes methods and false discovery rates for microarrays. *Genet Epidemiol* **23**: 70–86.

Gaasterland T, Bekiranov S. 2000. Making the most of microarray data. *Nature Genet* **24**: 204–206.

Golub TR, Slonim DK, Tamayo P, *et al.* 1999. Molecular classification of cancer: class discovery and class prediction by gene expression monitoring. *Science* **285**: 531–537.

Gutgemann A, Golob M, Muller S, Buettner R, Bosserhoff AK. 2001. Isolation of invasion-associated cDNAs in melanoma. *Arch Dermatol Res* **293**: 283–290.

Han GM, Chen SL, Shen N, Ye S, Bao CD, Gu YY. 2003. Analysis of gene expression profiles in human systemic lupus erythematosus using oligonucleotide microarray. *Genes Immun* **4**: 177–186.

Hoffmann R, Seidl T, Dugas M. 2002. Profound effect of normalization on detection of differentially expressed genes in oligonucleotide microarray data analysis. *Genome Biol* **3**: 0033.1–0033.11.

Johnston SR, Saccani-Jotti G, Smith IE, *et al.* 1995. Changes in estrogen receptor, progesterone receptor, and pS2 expression in tamoxifen-resistant human breast cancer. *Cancer Res* **55**: 3331–3338.

Kihara C, Tsunoda T, Tanaka T, *et al.* 2001. Prediction of sensitivity of esophageal tumors to adjuvant chemotherapy by cDNA microarray analysis of gene-expression profiles. *Cancer Res* **61**: 6474–6479.

Ladanyi M, Chan WC, Triche TJ, Gerald WL. 2001. Expression profiling of human tumors: the end of surgical pathology. *J Mol Diagn* **3**: 92–97.

Leygue E, Dotzlaw H, Watson PH, Murphy LC. 2000. Altered expression of estrogen receptor-$\alpha$ variant messenger RNAs between adjacent normal breast and breast tumor tissues. *Breast Cancer Res* **2**: 64–72.

Liu WM, Mei R, Di X, *et al.* 2002. Analysis of high density expression with signed-rank call algorithms. *Bioinformatics* **18**: 1593–1599.

Mariadason JM, Arango D, Corner GS, *et al.* 2002. A gene expression profile that defines colon cell maturation *in vitro*. *Cancer Res* **62**: 4791–4804.

Maxwell DT, Jacobson JD, King A, Chan PJ. 2002. Effect of pentoxifylline on tumor suppressor and proto-oncogene apoptosis in sperm. *J Assist Reprod Genet* **19**: 279–283.

Mycko MP, Papoian R, Boschert U, Raine CS, Selmaj KW. 2003. cDNA microarray analysis in multiple sclerosis lesions: detection of genes associated with disease activity. *Brain* **126**: 1048–1057.

Notterman DA, Alon U, Sierk AJ, Levine AJ. 2001. Transcriptional gene expression profiles of colorectal adenoma, adenocarcinoma, and normal tissue examined by oligonucleotide arrays. *Cancer Res* **61**: 3124–3130.

Okabe S, Fujimoto N, Sueoka N, Suganuma M, Fujiki H. 2001. Modulation of gene expression by (–) -epigallocatechin gallate in PC-9 cells using a cDNA expression array. *Biol Pharm Bull* **24**: 883–886.

Pfaffl MW, Wittmann SL, Meyer HHD, Bruckmaier RM. 2003. Gene expression of immunologically important factors in blood cells, milk cells and mammary tissue of cows. *J Dairy Sci* **86**: 538–545.

Ramaswamy S, Ross KN, Lander ES, Golub TR. 2003. A molecular signature of metastasis in primary solid tumors. *Nature Genet* **33**: 49–54.

Robinson P, White AC, Lewis DE, *et al.* 2002. Sequential expression of the neuropeptides substance P and somatostatin in granulomas associated with murine cysticercosis. *Infect Immun* **70**: 4534–4538.

Rus V, Atamas SP, Shustova V, *et al.* 2002. Expression of cytokine- and chemokine-related genes in peripheral blood mononuclear cells from lupus patients by cDNA array. *Clin Immunol* **102**: 283–290.

Schageman JJ, Basit M, Gallardo TD, Garner HR, Shohet RV. 2002. MarC-V: a spreadsheet-based tool for analysis, normalization and visualization of single cDNA microarray experiments. *Biotechniques* **32**: 338–344.

Siegel S. 1956. *Non-parametric Statistics for Behavioral Scientists.* McGraw-Hill: Maidenhead; 75–83.

Smid-Koopman E, Blok LJ, Chadha-Ajwani S, *et al.* 2000. Gene expression profiles of human endometrial cancer samples using

Copyright © 2004 John Wiley & Sons, Ltd. 

a cDNA-expression array technique: assessment of an analysis method. *Br J Cancer* **83**: 246–251.

SPSS for Windows, SPSS Inc., Chicago, IL, USA.

Su AI, Welsh JB, Sapinoso LM, *et al*. 2001. Molecular classification of human carcinomas by use of gene expression signatures. *Cancer Res* **61**: 7388–7393.

Tamayo P, Slonim D, Mesirov J, *et al*. 1999. Interpreting patterns of gene expression with self-organizing maps: methods and application to hematopoietic differentiation. *Proc Natl Acad Sci USA* **96**: 2907–2912.

Tanaka TS, Jaradat SA, Lim MK, *et al*. 2000. Genome-wide expression profiling of mid-gestation placenta and embryo using a 15 000 mouse developmental cDNA microarray. *Proc Natl Acad Sci USA* **97**: 9127–9132.

Thomas JG, Olson JM, Tapscott SJ, Zhao LP. 2001. An efficient and robust statistical modeling approach to discover differentially expressed genes using genomic expression profiles. *Genome Res* **11**: 1227–1236.

Troyanskaya OG, Garber ME, Brown PO, Botstein D, Altman RB. 2002. Non-parametric methods for identifying differentially expressed genes in microarray data. *Bioinformatics* **18**: 1454–1461.

Tusher VG, Tibshirani R, Chu G. 2001. Significance analysis of microarrays applied to the ionizing radiation purpose. *Proc Natl Acad Sci USA* **98**: 5116–5121.

Wang K, Gan L, Jeffery E, *et al*. 1999. Monitoring gene expression profile changes in ovarian carcinomas using cDNA microarray. *Gene* **229**: 101–108.

Wang W, Marsh S, Cassidy J, McLeod HL. 2001. Pharmacogenomic dissection of resistance to thymidylate synthase inhibitors. *Cancer Res* **61**: 5505–5510.

Wilcoxon F. 1945. Individual comparisons by ranking methods. *Biometric Bull* **1**: 80–83.

Wilcoxon F. 1947. Probability tables for individual comparisons by ranking methods. *Biometrics* **3**: 119–122.

Xu Y, Selaru FM, Yin J, *et al*. 2002. Artificial neural networks and gene filtering distinguish between global gene expression profiles of Barrett's esophagus and esophageal cancer. *Cancer Res* **62**: 3493–3497.

Yu CL, Sun KH, Tsai CY, Hsieh SC, Yu HS. 2001. Anti-dsDNA antibody upregulates interleukin 6, but not cyclo-oxygenase, gene expression in glomerular mesangial cells: a marker of immune-mediated renal damage. *Inflamm Res* **50**: 12–18.

Zhao Y, Pan W. 2003. Modified non-parametric approaches to detecting differentially expressed genes in replicated microarray experiments. *Bioinformatics* **19**: 1046–1054.

Zhou X, Tan FK, Xiong M, *et al*. 2001. Systemic sclerosis (scleroderma): specific autoantigen genes are selectively overexpressed in scleroderma fibroblasts. *J Immunol* **167**: 7126–7133.

Copyright © 2004 John Wiley & Sons, Ltd. 
